# Serum tumor necrosis factor-alpha concentrations are negatively correlated with serum 25(OH)D concentrations in healthy women

**DOI:** 10.1186/1476-9255-5-10

**Published:** 2008-07-24

**Authors:** Catherine A Peterson, Mary E Heffernan

**Affiliations:** 1 Department of Nutritional Sciences, University of Missouri-Columbia, Columbia, MO, 65211, USA

## Abstract

**Background:**

Circulating 25 hydroxyvitamin D (25 (OH)D), an accurate measure of vitamin D status, is markedly greater in individuals with increased exposure to ultraviolet B (UVB) light via sunlight or the use of artificial UV light. Aside from the known relationship between vitamin D and bone, vitamin D has also been implicated in immune function and inflammation. Furthermore, a mass of evidence is accumulating that vitamin D deficiency could lead to immune malfunction. Our overall objective was to study the relationship between vitamin D status (as determined by serum 25(OH) D concentrations) and inflammatory markers in healthy women.

**Methods:**

This observational study included 69 healthy women, age 25–82 years. Women with high UVB exposure and women with minimal UVB exposure were specifically recruited to obtain a wide-range of serum 25(OH)D concentrations. Health, sun exposure and habitual dietary intake information were obtained from all subjects. Body composition was determined by dual-energy-x-ray absorptiometry. A fasting blood sample was collected in the morning and analyzed for serum 25(OH)D, parathyroid hormone (iPTH), estradiol (E_2_), cortisol, and inflammatory markers [tumor necrosis factor -alpha (TNF-α), interleukin-6 and -10 (IL-6, IL-10), and C-reactive protein (CRP)].

**Results:**

Women with regular UVB exposure (Hi-D) had serum 25(OH)D concentrations that were significantly higher (*p *< 0.0001) and iPTH concentrations that were significantly lower (*p *< 0.0001) than women without regular UVB exposure (Lo-D). Although IL-6, IL-10, and CRP did not have a statistically significant relationship with 25(OH)D concentrations, linear regression models revealed a significant inverse relationship between serum 25(OH)D and TNF-α concentrations. This relationship remained significant after controlling for potential covariates such as body fat mass, menopausal status, age, or hormonal contraceptive use.

**Conclusion:**

Serum 25(OH)D status is inversely related to TNF-α concentrations in healthy women, which may in part explain this vitamin's role in the prevention and treatment of inflammatory diseases. Results gleaned from this investigation also support the need to re-examine the biological basis for determining optimal vitamin D status.

## Background

Circulating 25 hydroxyvitamin D (25(OH)D), an accurate measure of vitamin D status, is markedly increased in individuals who receive regular exposure to ultraviolet B (UVB) light via sunlight or the use of artificial UV light (such as tanning beds) [1-6]. Serum 25(OH)D is hydroxylated in the kidney, as well as in numerous other tissues, to its active form, 1,25-dihydroxyvitamin D (1,25(OH)_2_D). 1,25(OH)_2_D binds to nuclear vitamin D receptors in tissues throughout the body. Active vitamin D is responsible for maintaining calcium homeostasis primarily by increasing the efficiency of intestinal calcium absorption and by stimulating the differentiation of bone-resorbing osteoclasts. Furthermore, vitamin D deficiency increases secretion of parathyroid hormone, which accelerates bone breakdown and can lead to decreased bone formation and density [7,8].

There is a growing body of data supporting the contention that desirable serum 25(OH)D concentrations in healthy individuals need to be set higher than the current values to attain the optimal health benefits of vitamin D [8-11], especially the benefits beyond calcium homeostasis [12-14]. For no system does this ring truer than for the influence of vitamin D status on the immune system.

In the last few years, there has been an effort to understand the possible noncalcemic (i.e. non-calcium regulatory) roles of vitamin D, including its role in the immune system [15,16]. Most of the known biological effects of 1,25(OH)_2_D are mediated through the vitamin D receptor (VDR); and, within the immune system, the VDR is found in significant concentrations in the T lymphocyte and macrophage populations [16]. Moreover, the enzyme responsible for the final and rate-limiting hydroxylation step in the synthesis of active vitamin D, 25(OH)D-1-a-hydroxylase, is expressed by activated macrophages, allowing these phagocytic cells to synthesize and secrete 1,25(OH)_2_D in a regulated fashion [17]. Additionally, the major 1,25(OH)_2_D degrading enzyme, 24-hydroxylase, is also expressed in monocytes/macrophages [18]. All of these findings, then, suggest a paracrine role for vitamin D in the immune system [19].

Evidence is accumulating that vitamin D deficiency may lead to immune dysregulation. The relationship between low serum 25(OH)D concentrations and autoimmune disease (especially multiple sclerosis, Type I diabetes and rheumatoid arthritis) has been appreciated for some time [5,20,21]. More recently, studies have shown defective macrophage function, such as impaired chemotaxis, phagocytosis, and increased production of proinflammatory cytokines, in vitamin D-insufficiency [18]. Vitamin D has also been shown to downregulate the expression of monocyte toll-like receptors (TLRs), known inducers of inflammation that can prompt autoimmune disease exacerbation or sepsis [22]. In 2006, a double-blind, randomized, placebo-controlled trial showed that vitamin D supplementation improved cytokine profiles in patients with congestive heart failure [12].

Several provocative reports have been published that also support a role for vitamin D in reducing the risk of certain infectious diseases [23,24], in part through the induction of calthelcidin (also known as hCAP18, LL-37 and FALL-39), an antimicrobial polypeptide [25]. For example, in two seminal papers, Liu *et al *demonstrated that poor vitamin D status may increase susceptibility to *Mycobacterium tuberculosis *infection by inefficiently supporting the induction of cathelcidin mRNA in monocytes [26,27].

On balance, the published literature supports the need for further inquiry into vitamin D status and its immune system implications. Thus, our overall objective was to study the relationship between vitamin D status (as determined by serum 25(OH) D concentrations) and inflammatory markers in healthy women. Women with high UVB exposure and women with minimal UVB exposure were specifically recruited to obtain a wide-range of serum 25(OH)D concentrations [1,6,28] We hypothesized that serum 25(OH)D concentrations would be inversely correlated with circulating concentrations of inflammatory markers.

## Methods

### Subject volunteers

This study used an observational, cross-sectional design to explore the relationship between serum 25(OH)D concentrations and inflammatory marker concentrations in healthy women. Ethical approval for this study was received by the University of Missouri Health Sciences Institutional Review Board (Project number 1069397).

Volunteers were recruited from the University of Missouri-Columbia campus and surrounding community via email notices and flyers posted on campus bulletin boards, and at local tanning salons, fitness and community centers. To be included in the study, volunteers had to be Caucasian females who were at least 25 years of age. High vitamin D women (Hi-D) had to have used a broad-spectrum tanning bed at least once per week for a minimum of four months. Low vitamin D women (Lo-D) had minimal daily sunlight exposure, as assessed by a screening questionnaire, and did not use tanning beds. Volunteers were excluded from the study if they: took a vitamin D supplement other than a regular multivitamin; had a current or previous medical condition or took a medication affecting vitamin D status; had a current or previous medical condition or took a medication affecting immune function; had implanted metal that would interfere with the dual energy x-ray absorptiometry (DXA) scan; were undergoing ultraviolet radiation as medical therapy; exclusively used high-pressure (UVA-only) tanning beds; exercised more than 7 hours per week; were pregnant; or smoked cigarettes.

Following an initial screening for inclusion and exclusion criteria and after obtaining informed written consent, qualified volunteers were scheduled for testing. Subjects were instructed to refrain from exercise and to fast (water only) for 8 to 10 hours prior to their scheduled morning visit. On testing day, all subjects of childbearing age took a urine pregnancy test to confirm non-pregnant status. All study visits were conducted between late January and early June of 2007, the predicted seasonal nadir of solar UVB-produced serum 25(OH)D concentrations in mid-Missourians [29].

### Questionnaires and body composition

Four questionnaires were administered to all subjects: a one-page health history and medical questionnaire developed for this study to collect data on previous health conditions or diseases, menopausal status, current or previous medication use, and exercise habits; a one-page sun exposure questionnaire developed for this study to assess tanning bed use, outdoor sun exposure, and sunscreen use; a Fitzpatrick skin typing questionnaire, a well-established method of determining skin pigmentation and response to UVB exposure and thus potential for the photosynthesis of vitamin D in the skin [30]; and, the 88-question, self-administered Harvard Semi-quantitative Food Frequency Questionnaire, a validated tool to assess habitual dietary intake [31].

Body mass was measured without shoes to the nearest 0.1 kg and height to the nearest 0.5 cm using a medical balance beam scale. Body fat and lean body mass were measured by dual energy x-ray absorptiometry (DXA, Hologic Delphi A bone densitometer, Bedford, MA).

### Blood collection

All blood was drawn between the hours of 7:30 am and 11:30 am. Venous blood was collected into vacutainer tubes and allowed to clot at room temperature for 30 minutes. The coagulated blood was centrifuged; the serum was aliquoted into sterile microcentrifuge tubes, and stored at -80°C.

### Measurement of serum 25(OH)D

25(OH)D serum concentrations were measured using a ^125^I radioimmunoassay (RIA) kit (Diasorin, Stillwater, MN, Intra-assay CV = 10.8%). The 25(OH)D RIA is a two-step procedure. First, 25(OH)D and other hydroxylated metabolites are rapidly extracted from serum using acetonitrile. The extracted sample is then assayed using an antibody with specificity to 25(OH)D.

### Measurement of parathyroid hormone, estradiol and cortisol

Serum intact-PTH (iPTH) was measured using a commercially-available iPTH (1–84) enzyme-linked immunosorbent assay (ELISA) (ALPCO Diagnositics, Salem, NH, Intra-assay CV = 2.5%). Serum estradiol and cortisol were also measured using commercially available ELISA kits (ALPCO Diagnostics, Salem, NH, Intra-assay CV = 7.7% and 5.8%, respectively).

### Measurement of inflammatory markers

Four inflammatory markers were measured: IL-10, C-reactive protein (CRP), IL-6, and TNF-α. IL-10, IL-6, and TNF-α, were measured using commercially available high sensitivity ELISA kits (R&D Systems Inc., Minneapolis, MN, Intra-assay CV = 5.3%, 7.4%, and 7.7%, respectively). An ELISA was also used to measure CRP (R&D Systems Inc., Minneapolis, MN, Intra-assay CV = 5.5%).

### Statistical analysis

Unpaired two-tailed t-tests were used to determine differences in subject characteristics and measured outcomes; for data not normally distributed or of unequal variance, a rank-sum test was performed. Linear regression and univariate multiple regression models were used to determine the relationships between serum 25(OH)D and serum inflammatory markers. All statistics were performed using SAS statistical software version 9.1 (SAS Inc, Cary, NC). Statistical significance was accepted when P < 0.05.

## Results

### Vitamin D status

**Figure 1:**
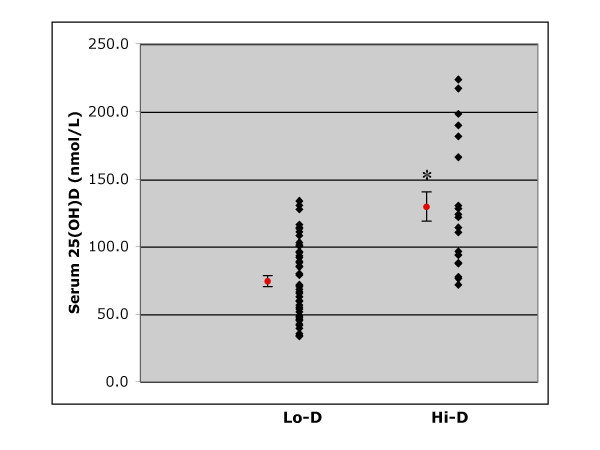
**Serum 25(OH)D concentrations of Lo-D and Hi-D status women**. Mean (± SEM) serum 25(OH)D concentrations of healthy women, age 25–82 years, categorized as low vitamin D status (Lo-D; n = 49) or high vitamin D status (Hi-D; n = 20) based on UVB exposure. Single points for each category are means (± SEMS). *Significantly different from Lo-D, *P *< 0.0001.

Serum 25(OH)D concentrations of all subjects are presented in Figure 1. Sixty-nine women between the ages of 25 and 82 years participated in the study. Forty-nine of the women were classified as Lo-D and 20 women were classified as Hi-D based on UVB exposure. The mean serum 25(OH)D status (nmol/L) of the Hi-D women (129.6 ± 11.0 nmol/L) was significantly higher than that of the Lo-D women (74.4 ± 4.0 nmol/L) (P < 0.0001).

### Subject characteristics and serum hormone concentrations

Subject characteristics and serum hormone concentrations by vitamin D status are presented in Table 1. There were no significant differences in age, height, weight, BMI, percent body fat, hormonal contraceptive use or serum estradiol or cortisol concentrations between vitamin D status groups. The mean iPTH concentration of the Hi-D women was significantly lower than that of the Lo-D women (P < 0.0001). Furthermore, there was a significant inverse relationship between 25(OH)D and iPTH concentrations (R^2 ^= 0.2498; P = 0.0001). The skin type of the Hi-D was significantly higher than that of the Lo-D group (P = 0.0031). The Fitzpatrick skin typing method determines skin type based on pigmentation and ability to burn and/or tan with sun exposure (Type I-IV, lighter-darker) [30]. Thus, it is not surprising that the Hi-D women had a higher-level skin type because their skin is capable of tanning; while women with lower-level skin types would not be expected to use a tanning bed since their skin is less able to tan. There were no differences between vitamin D status groups for dietary intakes of energy, macronutrients including omega-3 fatty acids, alcohol or caffeine (data not shown).

**Table 1:** Subject characteristics and serum hormone concentrations.

**Characteristic/Hormone**	**Lo-D (n = 49)**	**Hi-D (n = 20)**	**P Value**
**Age (years)**	39.8 ± 1.8	41.7 ± 3.5	0.5733
**Height (m)**	1.70 ± 0.01	1.65 ± 0.01	0.6894
**Weight (kg)**	65.9 ± 1.6	67.9 ± 2.7	0.3800
**Body Mass Index (kg/m^2^)**	23.8 ± 0.5	25.0 ± 1.1	0.2488
**Body Fat (%)**	30.1 ± 1.0	30.6 ± 1.7	0.7665
**Skin Type**	2.4 ± 0.1	3.1 ± 0.2*	0.0031
**Contraceptive Use (%)**	31%	20%	0.3780
**Estradiol (pg/mL)**	158.0 ± 15.6	151.3 ± 15.3	0.7974
**Cortisol (μg/dL)**	8.4 ± 0.5	9.4 ± 1.2	0.3129
**iPTH (pg/mL)**	48.1 ± 3.1	26.2 ± 2.6*	<0.0001

Subject characteristics and serum hormone concentrations of healthy women, age 25–82 years, categorized as low vitamin D status (Lo-D) or high vitamin D status (Hi-D) based on UVB exposure. Data are expressed as means ± SEM. *Significantly different from Lo-D, *P *< 0.05.

### Inflammatory marker outcomes

Mean serum TNF-α was significantly lower in the Hi-D than the Lo-D women (1.22 ± 0.11 vs. 0.79 ± 0.11, P = 0.0200. IL-10, CRP and IL-6 did not significantly differ between groups.

**Figure 2:**
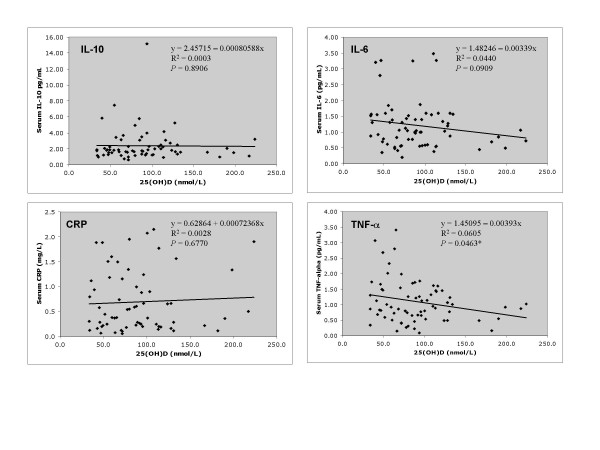
**The relationship between serum 25(OH)D concentrations and inflammatory marker concentrations**. The relationship between serum 25(OH)D concentrations and serum IL-10, C-reactive protein (CRP), IL-6 and TNF-a concentrations in healthy women, ages 25–82 years (n = 69). Linear regression equations for each inflammatory marker are shown. * Slope of regression line significantly less than zero, P < 0.05.

Figure 2 shows the relationships between 25 (OH)D and IL-10, CRP, IL-6, and TNF-α. Serum 25(OH)D concentrations were negatively correlated with TNF-α (R^2 ^= 0.0605, P = 0.0463). Thus, serum 25(OH)D status explained 6.05% of the variation in TNF-α concentrations. IL-10, CRP and IL-6 concentrations were not significantly associated with the concentration of 25(OH)D in serum.

When controlling for percent body fat, menopausal status, age, serum estradiol, serum cortisol, and hormonal contraceptive use, a significant relationship (P < 0.05) remained between 25(OH)D and TNF-α. Controlling for percent body fat, menopausal status, age, serum estradiol, serum cortisol, and hormonal contraceptive use did not change the relationship between 25(OH)D concentrations and IL-6, IL-10, and CRP. Analysis of potential covariates revealed a significant positive association between age and IL-6 (R^2 ^= 0.09413, P = 0.0116); and menopausal status and IL-6 (R^2 ^= 0.0764, P = 0.0246).

## Discussion

The objective of the present study was to determine the relationship between 25(OH)D concentrations and inflammatory marker concentrations in healthy women. Although IL-6, IL-10, and CRP did not have a statistically significant relationship with 25(OH)D concentrations, linear regression models revealed a significant inverse relationship between serum 25(OH)D and serum TNF-α concentrations. This relationship remained significant after controlling for potential covariates such as body fat mass, menopausal status, age, or hormonal contraceptive use. Hinton *et al*. found that hormonal contraceptive use was associated with greater TNF-α concentrations in young female athletes [32]. Our data from healthy female non-athletes representing a much wider age range did not reveal such a relationship with TNF-α (*P *= 0.2336); however, like Hinton *et al*., there was a significant relationship between hormonal contraceptive and serum cortisol level (*P *= 0.0030). Interestingly, in our study serum 25(OH)D remained a significant predictor of TNF-α even after controlling for contraceptive use and cortisol concentrations. The lack of significance between serum estradiol and any of the inflammatory markers (data not shown) supports previous research indicating that, in premenopausal women, menstrual phase may affect circulating cytokine concentrations, but the impact is generally not detectable [33].

TNF-α is produced by numerous cell types, including macrophages, monocytes, T-cells, smooth muscle cells, adipocytes, and fibroblasts [34] many of which also have VDR [14,15,35]. Thus, it is difficult to discern the specific mechanisms by which elevations in systemic 25(OH)D attenuate circulating TNF-α concentrations. Nonetheless, our results agree with experimental data showing that vitamin D is capable of suppressing TNF-α production [36-39]. Zhu *et al*. recently showed that in the colonic tissue of mice with inflammatory bowel disease, 1,25(OH)_2_D was capable of down-regulating several genes associated with TNFα, including proteins involved in the transcription of TNFα, one of its primary receptors, and TNF-α itself [39].

Human studies of diseased populations have also shown beneficial effects of vitamin D status on TNF-α concentrations. Serum concentrations of TNF-α increased in unsupplemented congestive heart failure patients over a period of 9 months, whereas serum TNF-α concentrations in patients receiving daily supplementation of vitamin D (2000 IU) remained constant [12]. Calcitriol (1,25(OH)_2_D_3_) supplementation for 6 months in post-menopausal women with osteoporosis resulted in a significant reduction in serum TNF-α concentrations and an increase in bone mineral density [40]. Additionally, six months of calcitriol supplementation in hemodialysis patients also caused significant decreases in serum TNF-α [41]. Our study is the first to show a significant inverse relationship between serum 25(OH)D and TNF-α concentration in a healthy population.

TNF-α concentrations are increased in several disease states such multiple sclerosis (MS), inflammatory bowel disease (IBD), rheumatoid arthritis (RA), heart disease, and osteoporosis; and are often correlated with clinical impairment [42,43]. Therefore, attenuating the concentrations of circulating TNF-α has the potential to positively impact the risk for or treatment of such conditions. Our data suggest that serum 25(OH)D status explains ~6% of the variation in TNF-α concentrations in healthy women, thus a mild relationship.

Even a slight drop in circulating TNF-α due to improved vitamin D status may have clinical significance. MS patients with < 2 active brain lesions visible on magnetic resonance imagery were shown to have serum TNF-α concentrations that were slightly but significantly (1.6 pg/mL) less than those with ≥ 2 active brain lesions [44]. Patients with active ulcerative colitis were found to have 41% greater mean TNF-α concentrations than those with inactive disease (9.46 and 5.54 pg/mL, respectively); while, those with active Crohn's disease had TNF-α concentrations that were only 18% greater than patients with inactive Crohn's (14.0 and 11.5 pg/mL, respectively) [45].

Increases in circulating TNF-α concentrations have been associated with heart disease progression. Koller-Strametz reported that TNF-α concentrations were 3.2 ± 0.2 pg/mL in patients with New York Heart Association (NYHA) function class II, 4.0 ± 0.3 pg/mL in NYHA function class III patients, and 5.3 ± 0.9 pg/mL in NYHA function class IV patients [46].

Anti-TNF-α medications are efficacious in the management of IBD [47]. Martinez-Borra *et al*. found that patients with lower TNF-α concentrations (14 ± 25 pg/mL) prior to treatment with the anti-TNF drug, infliximab, responded to the treatment, whereas non-responders had significantly higher baseline serum concentrations (201 ± 362 pg/mL) [48]. Therefore, it is possible vitamin D supplementation may be a viable adjunct to anti-TNF therapy.

Human studies involving diseased populations have shown positive relationships between 25(OH)D concentrations and IL-10 [12]. Despite this evidence, in the present study, serum IL-10 was not significantly correlated with serum 25(OH)D, suggesting that in healthy adults, vitamin D status does not affect IL-10 secretion into systemic circulation.

Similarly, serum 25(OH)D and serum CRP were not correlated in the present study. As a non-specific inflammatory marker of general wellness, CRP increases with mild chronic infection, aging, and tissue damage [49]. Research in diseased populations, such as diabetes [50], arthritis [51,52], prolonged chronic illness [53], and clinical vitamin D deficiency (25(OH)D <27.5 nmol/L) [54] have demonstrated negative associations between vitamin D status and CRP concentrations. Nevertheless, intervention studies of healthy post-menopausal women [55] and patients with congestive heart failure [12] failed to see changes in CRP concentrations after vitamin D supplementation.

Although the result of the linear regression analysis was not statistically significant, there appears to be a slight tendency towards an inverse relationship between 25(OH)D concentrations and serum IL-6 (P = 0.0909). Several *in vitro *studies have shown that 1,25(OH)_2_D and several of its analogs are capable of inhibiting the production of IL-6 in various cell types [38,56-59]; while most published *in vivo *studies have failed to show an effect of vitamin D status on circulating IL-6 concentrations in humans [12,52,60,61]. One report, however, involving hemodialysis patients with elevated parathyroid hormone (PTH) demonstrated that both oral and intravenous 1,25(OH)_2_D supplementation were capable of significantly decreasing serum IL-6 concentrations following 6 months of treatment [62]. It has been well documented that PTH induces the production of IL-6 by osteoblasts [63,64], thus, it is likely that the effects of vitamin D supplementation on serum IL-6 in this population were mediated primarily through the inverse relationship between 25(OH)D and PTH. In our study, there was no relationship between intact PTH and IL-6 concentrations (P = 0.8039). The significant relationship found between age and IL-6 (P = 0.0116) in this study was anticipated due to several reports showing that circulating IL-6 concentrations increase with advancing age [65-68]. Further, IL-6 has been implicated in age-associated diseases (such as lymphoproliferative disorders, multiple myeloma, osteoporosis, and Alzheimer's disease) and frailty; and, it is postulated that certain clinically important late-life changes are due to an inappropriate presence of IL-6. Therefore, our results indicating a trend for a negative relationship between vitamin D status and IL-6 concentrations warrants further investigation. The lowering of circulating IL-6 through the improvement of vitamin D nutriture may have the potential to decrease disability and mortality in older populations in addition to helping maintain muscle strength and bone health.

The range of serum 25 (OH)D concentrations observed in our healthy female subjects are in accordance with the overwhelming number of reports documenting the prevalence of vitamin D deficiency and insufficiency in the general population [69-75]. In recent years, mounting data has highlighted the need to re-examine vitamin D status and recommendations [76]. Bischoff-Ferrari *et al*. summarized results from randomized controlled trials, prospective and cross-sectional epidemiologic studies, strong mechanistic evidence, and dose-response relationships to determine an optimal serum 25(OH)D concentration [77]. They showed that for all endpoints (bone mineral density, lower-extremity function, dental health, and risk of falls, fractures, and colorectal cancer), optimal 25(OH)D status began at 75 nmol/L. Our study demonstrates that like these other health outcomes, circulating TNF-α concentrations continue to be associated with serum 25(OH)D concentrations above this point, in a manner consistent with decreased disease risk/progression (i.e. lower TNF-α concentrations).

The primary limitation of this study was sample size. Women who were regularly exposed to UVB light and qualified to participate based on our inclusion and exclusion criteria were far more difficult to recruit than women with minimal UVB exposure. Additionally, women who tan regularly are inherently different from non-tanners. Frequent tanning bed use is associated with high risk behaviours, including frequent dieting, laxative use or vomiting to control weight, cigarette smoking, binge drinking, and recreational drug use. [78]. In light of this, the present study was designed to control or account for these behaviors through subject inclusion/exclusion criteria and inclusion of pertinent questionnaire data in the multiple regression analysis.

## Conclusion

Serum TNF-α concentrations are negatively correlated with vitamin D status in healthy women. This study is the first known report to show this inverse relationship in a non-diseased population. Results gleaned from this investigation also support the need to re-examine the biological basis for determining optimal vitamin D status. More studies are needed to fully characterize the relationship between vitamin D and TNF-α relationship; but if proven effective, vitamin D therapy may show promise as adjunct to anti-TNF therapy in inflammatory disease states.

## Abbreviations

1,25(OH)_2_D: 1,25-dihydroxyvitamin D; 25(OH)D: 25-hydroxyvitamin D; CRP: C-reactive protein; DXA: dual-energy x-ray absorptiometry; Hi-D: high vitamin D status; Lo-D: low vitamin D status; IL-6: interleukin-6; IL-10: interleukin 10; TNF-α: tumor necrosis factor-alpha.

## Competing interests

The authors declare that they have no competing interests.

## Authors' contributions

CAP developed the project idea and study design; obtained IRB approval; and wrote the manuscript. MEH, the graduate student under CAP's mentorship, coordinated the project including subject recruitment, testing, sample collection and analyses. Both authors contributed to the final edits of the manuscript.
